# Single-molecule force spectroscopy of ligand–receptor mechanics at extracellular vesicle biointerfaces

**DOI:** 10.1039/d6tb01369d

**Published:** 2026-09-30

**Authors:** Sakurako Tani, Reed Jacobson, Sangdeuk Ha, Connor Edvall, Yagna P. R. Jarajapu, Mohiuddin Quadir, Sanku Mallik, Jiha Kim, Yongki Choi

**Affiliations:** a Department of Physics, North Dakota State University Fargo ND 58108 USA yongki.choi@ndsu.edu; b Department of Biological Sciences, North Dakota State University Fargo ND 58108 USA; c Department of Pharmaceutical Sciences, North Dakota State University Fargo ND 58108 USA; d Department of Coatings and Polymeric Materials, North Dakota State University Fargo ND 58108 USA; e Materials and Nanotechnology Program, North Dakota State University Fargo North Dakota 58108 USA; f Molecular and Cellular Biology Program, North Dakota State University Fargo ND 58108 USA

## Abstract

Extracellular vesicles (EVs) are nanoscale membrane-bound particles that present surface receptors for affinity capture, sensing, and targeted biointerface design. Although EV surface-marker expression is widely used to guide ligand selection, the molecular-scale rupture mechanics and force-dependent dissociation behavior of EV receptor–ligand interactions remain poorly understood. Here, atomic force microscopy-based single-molecule force spectroscopy and dynamic force spectroscopy are used to quantify ligand-accessible recognition, rupture-force distributions, and apparent Bell–Evans parameters for three pancreatic cancer-associated EV surface targets: integrins (ITG), epithelial cell adhesion molecule (EpCAM), and glypican-1 (GPC1). EVs derived from PANC-1 and hTERT–HPNE cells were compared as a model system. PANC-1 EVs exhibited higher particle concentrations and stronger ITG and GPC1 expression than HPNE EVs. AFM recognition maps showed markedly higher apparent ITG recognition and moderately higher GPC1 recognition on PANC-1 EVs, whereas EpCAM recognition was similar between the two EV preparations. Fixed-speed rupture-force distributions and loading-rate-dependent analysis revealed interaction-dependent differences in apparent rupture behavior. Across both EV preparations, EpCAM–antibody interactions tended to show higher apparent force-free off-rates than ITG–cRGD and GPC1–antibody interactions, while ITG and GPC1 were less clearly separated. Together with recognition mapping, these results demonstrate that ligand-accessible recognition and force-dependent rupture behavior provide complementary information about EV surface interactions.

## Abstract 2

AFM force spectroscopy distinguishes ligand accessibility from rupture mechanics on extracellular vesicles. EpCAM–antibody interactions tend to show higher apparent off-rates than ITG–cRGD and GPC1–antibody interactions.

## Graphical abstract

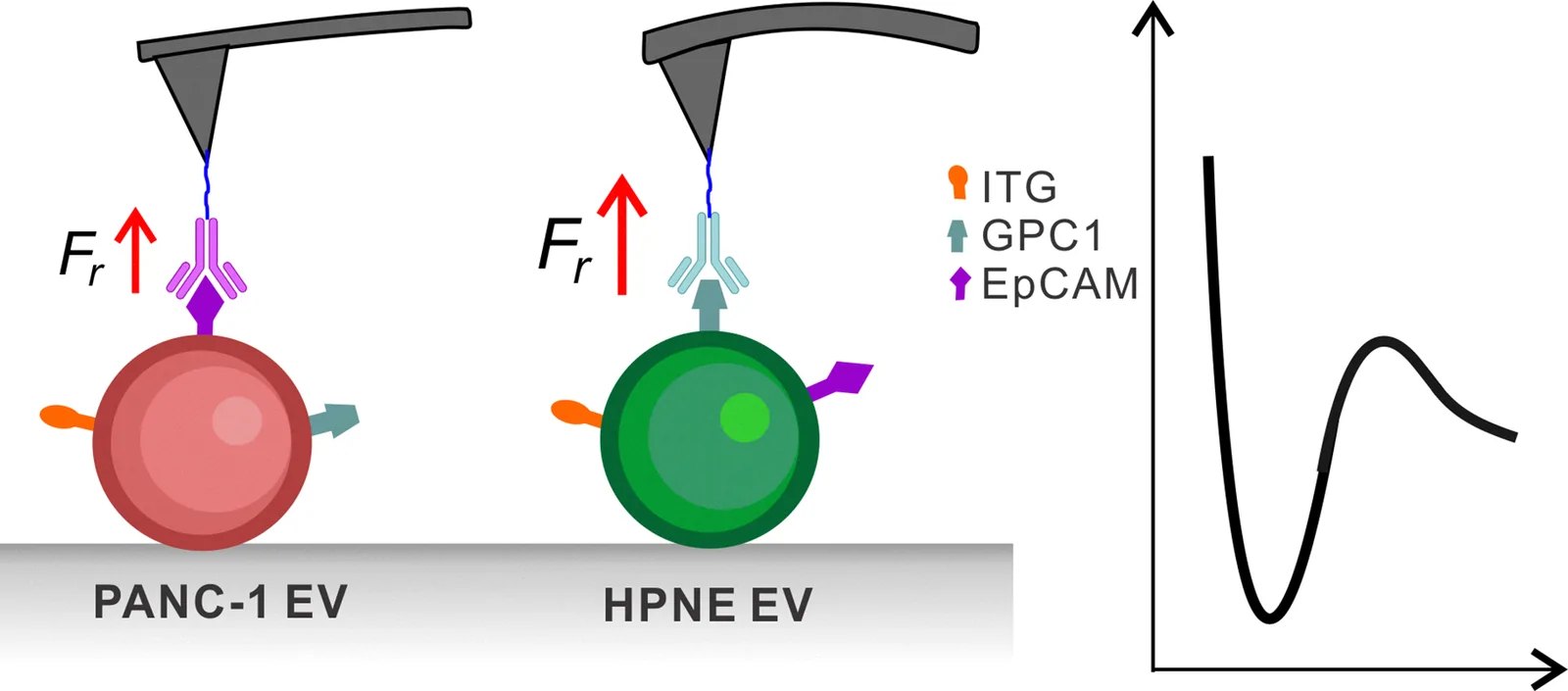

## Introduction

Extracellular vesicles (EVs) are lipid-enclosed nanoscale particles that transport proteins, nucleic acids, and lipids reflective of their parent cells. Because EVs are abundant in biofluids such as blood, saliva, and urine, they have attracted considerable interest as carriers of disease-associated molecular information and as targets for diagnostic, therapeutic, and bioengineering applications.^1–3^ In cancer, tumor-cell-derived EVs can exhibit altered membrane protein expression, oncogenic cargo, and cancer-associated mutations, motivating the development of EV capture and sensing platforms capable of selectively enriching disease-associated EV subpopulations from heterogeneous biological backgrounds.^4–10^

Many EV isolation and detection strategies rely on affinity interactions between EV surface receptors and immobilized ligands, including antibodies, peptides, and other receptor-binding molecules. Conventional assays such as enzyme-linked immunosorbent assay, flow cytometry, surface plasmon resonance, and nanoplasmonic sensors have provided important ensemble-level information on EV surface-marker expression and binding.^11–13^ However, engineered EV affinity interfaces also impose mechanical and kinetic constraints that are not fully captured by receptor abundance alone. During EV capture, washing, flow, repeated contact, or surface regeneration, ligand–receptor bonds can experience mechanical loading, and EV retention may depend on the force-dependent stability and dissociation kinetics of individual biomarker–ligand interactions. Thus, a quantitative understanding of molecular-scale rupture mechanics at EV biointerfaces can complement conventional expression-based profiling and inform the design of EV capture, sensing, and affinity-interface materials.

Single-molecule force spectroscopy (SMFS) using functionalized atomic force microscopy (AFM) probes provides a direct approach to quantify ligand–receptor rupture forces and dynamic dissociation behavior at membrane interfaces.^14^ By attaching ligands to an AFM probe through a flexible polymer linker, SMFS enables repeated formation and rupture of tethered ligand–receptor complexes and can map ligand-accessible recognition events on nanoscale membrane surfaces. Dynamic force spectroscopy (DFS), in which rupture forces are measured as a function of loading rate, further enables extraction of apparent force-dependent dissociation parameters using models such as Bell–Evans analysis. This approach is particularly useful for EVs because their compliant lipid membranes, high curvature, and absence of cytoskeletal support can influence the apparent mechanical response of receptor–ligand bonds under load.

Previously, we used AFM-based SMFS to characterize cyclic RGD (CRGDKGPDC; cRGD) interactions with integrin αvβ5 on pancreatic cancer cell membranes and showed that receptor density and multivalency strongly modulate the effective kinetics and stability of cRGD–integrin complexes.^15^ However, much less is known about how analogous ligand–receptor interactions behave on EV membranes, where receptors are embedded in a softer, cytoskeleton-free nanoscale vesicle environment. This distinction is important for EV-targeting biointerfaces because the apparent binding stability of EVs may depend not only on receptor expression but also on receptor accessibility, membrane compliance, ligand geometry, and loading-rate-dependent rupture behavior.

In this study, we focus on three widely studied pancreatic ductal adenocarcinoma (PDAC)-associated EV surface targets: integrins (ITGαvβ5, probed using cRGD), epithelial cell adhesion molecule (EpCAM), and glypican-1 (GPC1).^5,10,16,17^ These targets were selected because they are commonly investigated EV surface markers with distinct ligand-recognition chemistries and relevance to affinity capture, sensing, and EV biointerface design. Tumor-derived EV integrins have been linked to organotropic metastasis, and αvβ5 integrins can be targeted by cRGD ligands for affinity capture and delivery applications.^10^ EpCAM is a well-established epithelial and tumor-associated surface marker that is widely used for EV enrichment and capture.^16^ GPC1-positive EVs have also been explored as candidate pancreatic cancer-associated EV markers.^17^ Despite extensive studies of their expression and use in EV enrichment, the molecular-scale rupture mechanics and apparent dissociation behavior of these EV surface targets remain poorly understood.

Here, we combine EV characterization, AFM-based SMFS, and DFS to compare ligand-accessible recognition, rupture-force distributions, and apparent Bell–Evans parameters for ITG–cRGD, EpCAM–antibody, and GPC1–antibody interactions on EVs isolated from pancreatic cancer PANC-1 cells and non-transformed pancreatic ductal hTERT–HPNE cells. By comparing ensemble biomarker expression with single-vesicle recognition probabilities and loading-rate-dependent rupture behavior, we evaluate how receptor accessibility and apparent bond stability jointly define EV biomarker–ligand interactions at compliant nanoscale biointerfaces. We further compare ITG–cRGD mechanics on EV membranes with our previous measurements on intact pancreatic cancer cell membranes to assess how the EV membrane environment influences apparent force-dependent dissociation. Together, these measurements provide a mechanics-informed framework for evaluating EV biomarker–ligand interactions in capture, sensing, and affinity-interface design.

## Results and discussion

### EVs isolated from two pancreatic cell lines

EVs were isolated from PANC-1 and HPNE cell-conditioned media and characterized by morphology, size distribution, particle concentration, and EV-associated protein markers (Fig. 1). AFM and SEM images showed nanoscale vesicular particles with rounded morphology, consistent with surface-immobilized EVs (Fig. 1a–b). Nanoparticle tracking analysis (NTA) showed that both preparations contained particles primarily in the ∼100–200 nm diameter range, with a modal size near 140 nm (Fig. 1c). The total particle concentration was on the order of 10^11^ mL^−1^, with PANC-1 EV preparations showing a higher particle concentration than HPNE EV preparations under the isolation conditions used here. This higher particle concentration is consistent with previous reports that pancreatic cancer cells can release elevated levels of EVs compared with non-malignant pancreatic cells.^5,18^

**Fig. 1:**
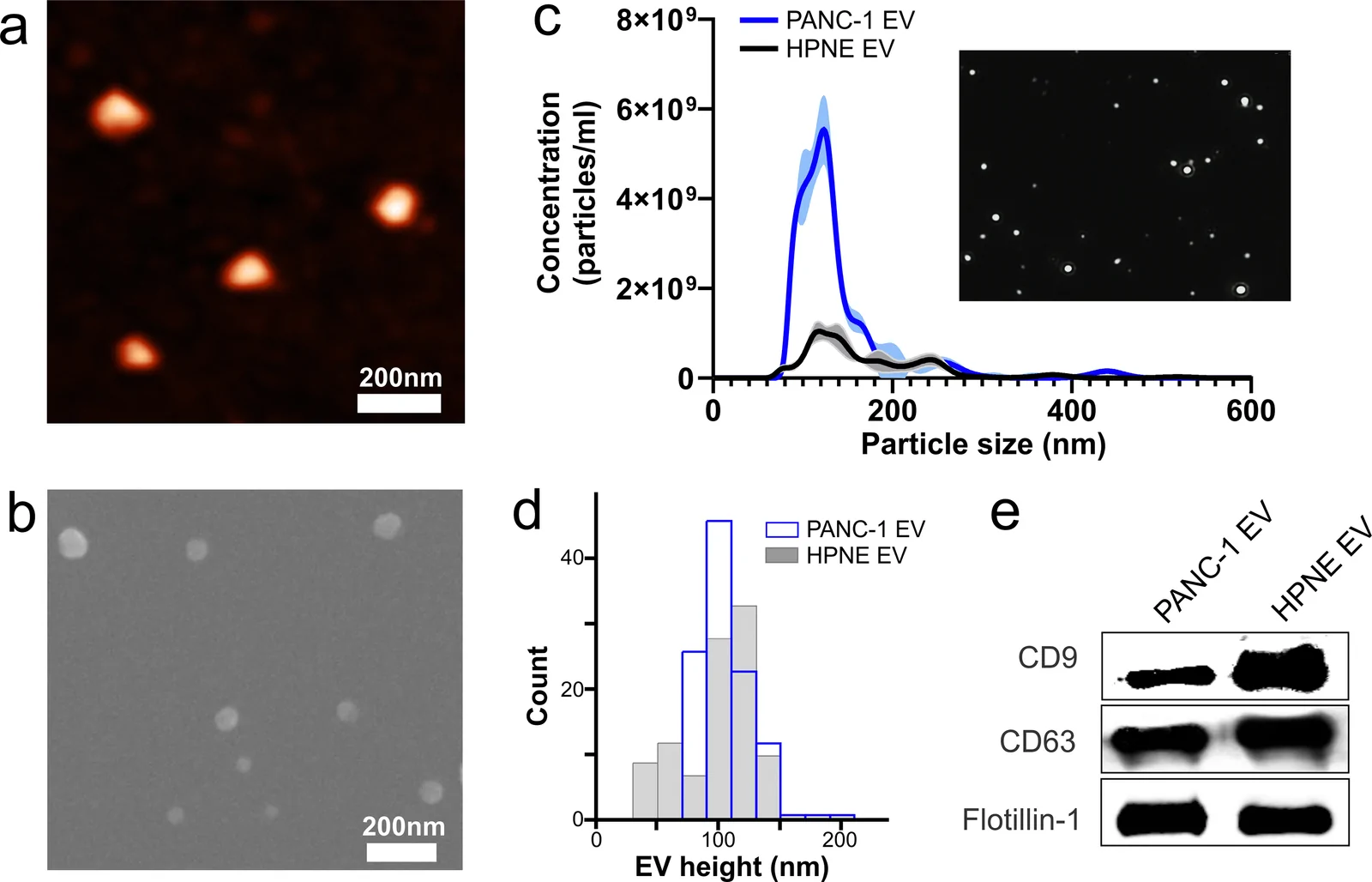
Characterization of EVs isolated from PANC-1 and HPNE cells. Representative (a) AFM and (b) SEM images of EVs isolated from PANC-1 cells. (c) Concentration and size distributions of PANC-1 EVs and HPNE EVs measured by nanoparticle tracking analysis (NTA). The inset shows a representative light-scattering image acquired during NTA measurement. (d) AFM height distributions of surface-immobilized PANC-1 EVs and HPNE EVs. (e) Immunoblots of EV-associated marker proteins CD9, CD63, and flotillin-1 in PANC-1 EVs and HPNE EVs.

AFM height analysis of surface-immobilized vesicles after drying under a nitrogen stream further confirmed nanoscale particle dimensions (Fig. 1d). These measurements characterize the deposited particles under ambient imaging conditions, while NTA reports their hydrodynamic diameters in suspension. Immunoblotting confirmed the presence of the EV-associated markers CD9, CD63, and flotillin-1 in both PANC-1 and HPNE EV preparations (Fig. 1e). Together, these morphological, size, and marker analyses confirm that the isolated vesicle preparations are suitable model EV samples for subsequent AFM-based ligand-recognition and force-spectroscopy measurements.

### Molecular recognition of EV biomarkers by single-molecule force spectroscopy

Molecular recognition between EV membrane biomarkers and their ligands was probed using AFM-based single-molecule force spectroscopy (SMFS) (Fig. 2). Fig. 2a and b schematically illustrates the experimental configuration. EVs derived from PANC-1 or HPNE cells were immobilized on poly-l-lysine (PLL)-coated substrates, and AFM probes were functionalized with either cRGD peptides for integrin recognition or antibodies for EpCAM and GPC1 recognition through flexible PEG linkers, using previously reported coupling strategies.^15,19,20^ AFM step-height measurements of identically prepared substrates under ambient conditions gave an apparent PLL thickness of 0.8 ± 0.3 nm (Fig. S1). Details are provided in the Experimental Section. Fluorescence imaging of functionalized silicon nitride substrates confirmed chemistry-dependent ligand coupling to the surface (Fig. S1). This configuration enables repeated approach–retraction cycles of a ligand-functionalized AFM probe over immobilized EVs, allowing ligand–receptor complexes to form and rupture under controlled mechanical loading.

**Fig. 2:**
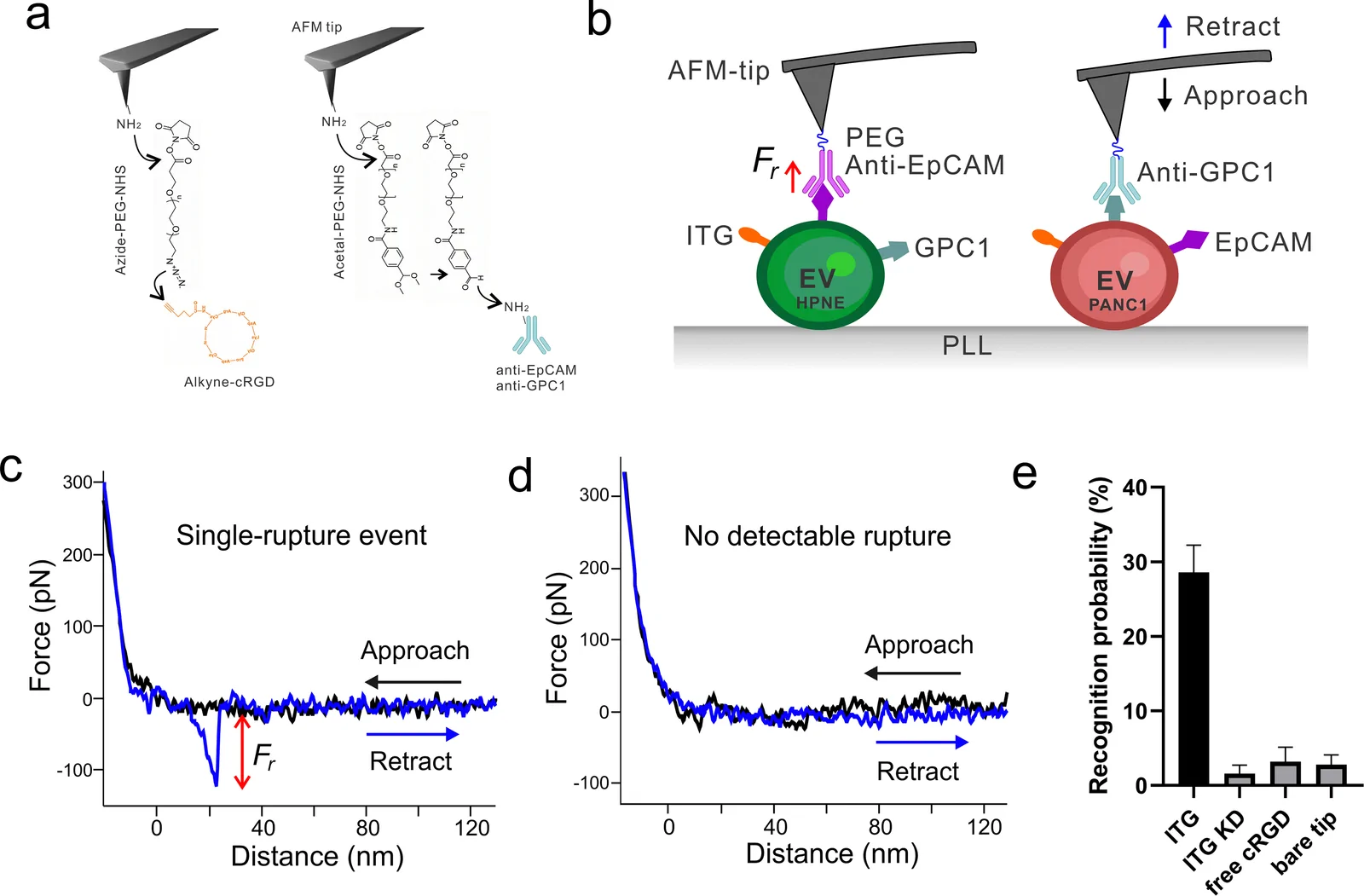
AFM-based single-molecule force spectroscopy of ligand–receptor interactions at EV biointerfaces. (a) Schematic of AFM probe functionalization with cRGD peptide or antibodies through PEG linkers. (b) Schematic of AFM–SMFS measurements in which ligand-functionalized AFM probes contact EVs immobilized on a PLL-coated substrate and are retracted to probe ligand–receptor rupture events. (c) Representative accepted force–distance curve showing a single, well-defined rupture event used for rupture-force analysis. (d) Representative no-binding force–distance curve showing overlapping approach and retraction traces. (e) Summary of specificity controls showing reduced apparent recognition probability after free-ligand blocking, bare-tip measurements, or ITG knockdown controls. Representative examples of excluded force–distance curves are provided in Fig. S2.

To quantify apparent ligand-specific rupture forces, the functionalized AFM probe was brought into contact with EV-coated substrates while monitoring cantilever deflection. During the approach segment, indentation was limited to approximately 5–10% of the apparent height of the immobilized EV to minimize additional probe-induced deformation during force measurement in PBS. The probe was then retracted along the *z*-axis while force–distance curves were recorded. Fig. 2c shows a representative accepted force–distance curve containing a single, well-defined rupture event. During retraction, selected curves displayed a short molecular extension followed by a discrete force drop, consistent with rupture of a ligand–receptor connection measured through the PEG linker and compliant EV membrane environment. In contrast, curves without detectable ligand–receptor rupture showed largely overlapping approach and retraction traces (Fig. 2d).

The extension preceding rupture can include contributions from PEG linker extension, receptor flexibility, receptor anchoring compliance, and local EV membrane deformation.^21,22^ Curves were evaluated using rupture position relative to contact, event morphology, baseline stability, and background-control behavior. Table S1 summarizes contact-to-rupture separations observed in representative accepted curves and does not define a PEG-contour-length cutoff. Curves showing multiple rupture steps, broad adhesive features, unusually long or tether-like extensions, unstable baselines, or complex rupture patterns were excluded from the single-rupture analysis. Here, single-rupture denotes one resolvable force drop. Representative examples of excluded curves are provided in Fig. S2.

Specificity was evaluated using bare-tip measurements, matched linker-only probes, competitive blocking with free ligand or antibody, and integrin-knockdown controls (Fig. 2e and Fig. S3). The competitive-blocking measurements retained the complete ligand/PEG-functionalized probe and therefore assessed residual interactions from the same probe chemistry after occupation of the corresponding EV binding sites. For ITG recognition, pre-incubation of PANC-1 EVs with excess free cRGD or the use of ITGαv-knockdown EVs reduced the apparent recognition probability from 28.6% to 3.2% and 1.6%, respectively, approaching the 2.8% bare-tip background. Matched azido–PEG_12_ and capped acetal–PEG control probes produced apparent event probabilities of 2.7% and 3.3%, respectively. Similarly, free-antibody blocking reduced EpCAM and GPC1 recognition from 10.6% and 18.8% to 2.7% and 3.7%, respectively. Together, the linker-only, competitive-blocking, and knockdown controls support assignment of the accepted events to the intended ligand–biomarker interactions.

The possible contribution of unresolved multiple-bond formation was estimated using a Poisson model. Taking the apparent recognition probability *p* as an estimate of *P*(*N* ≥ 1), the mean number of bonds per contact is *λ* = −ln(1 − *p*), and *P*(*N* ≥ 2) = 1 − *e*^−*λ*^(1 + *λ*). Across the six biomarker/EV conditions, the model predicts that 84.1–95.6% of interaction-positive contacts contain one bond, while 4.4–15.9% may contain two or more bonds (Table S2). The highest estimated multiple-bond fraction was obtained for PANC-1 ITG-cRGD, which also had the highest recognition probability. These estimates support predominantly single-bond-like events, consistent with the operational designation accepted single-rupture events.

### EV biomarker expression, ligand-accessible recognition, and fixed-speed rupture mechanics

Western blotting was used to compare ensemble biomarker expression, while SMFS assessed ligand-accessible recognition and fixed-speed rupture-force distributions for ITG, EpCAM, and GPC1 on PANC-1 and HPNE EVs (Fig. 3). Immunoblotting showed that PANC-1 EVs exhibited stronger ITGαv, ITGβ5, and GPC1 signals than HPNE EVs, whereas EpCAM was detectable in both EV preparations (Fig. 3a). These ensemble expression trends suggest that ITG and GPC1 are enriched on PANC-1 EVs relative to HPNE EVs in this model system and confirm the presence of all three targets for subsequent ligand-based recognition studies.

**Fig. 3:**
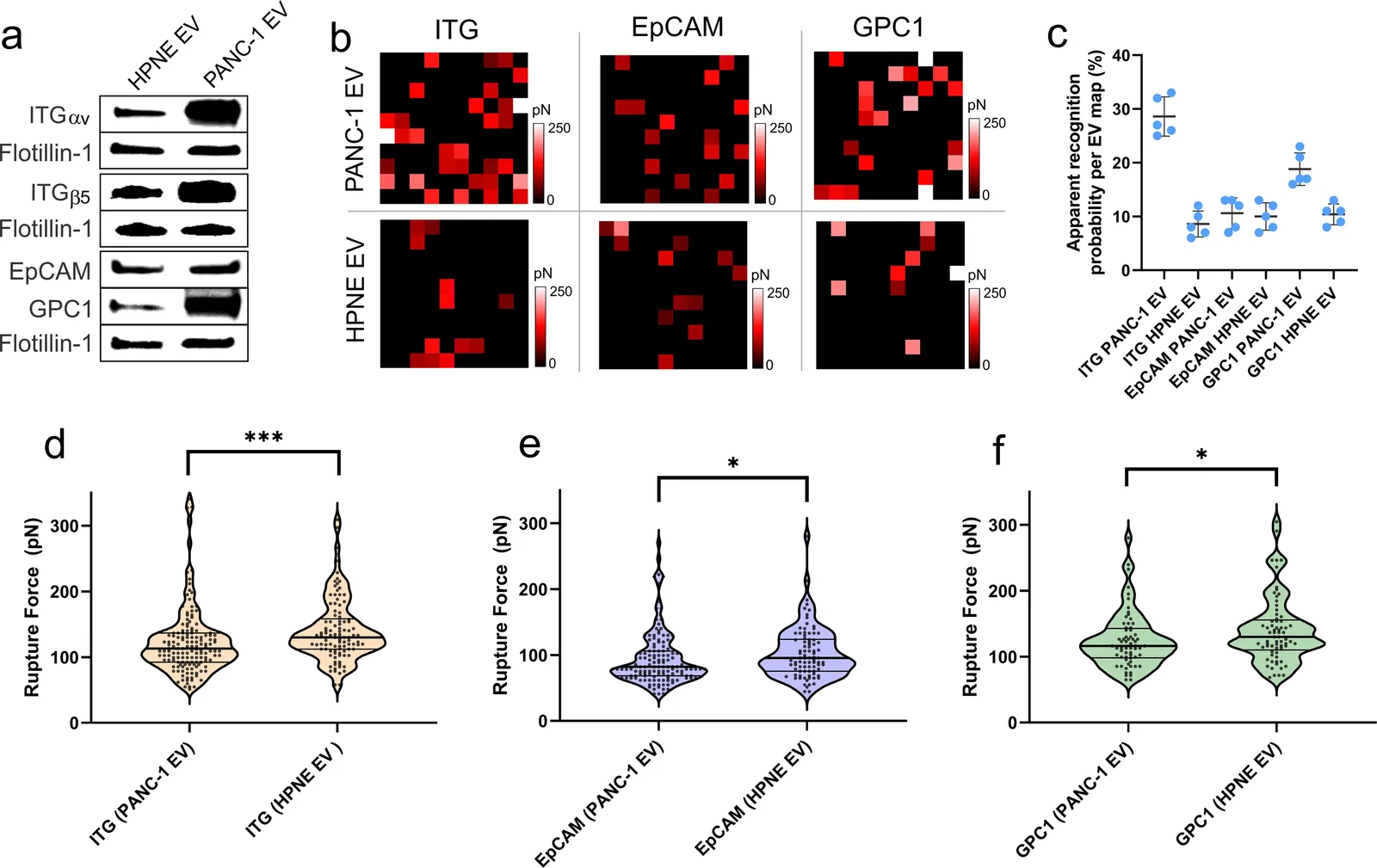
EV biomarker expression, ligand-accessible recognition, and fixed-speed rupture-force distributions. (a) Immunoblots of target EV surface biomarkers ITGαv, ITGβ5, EpCAM, and GPC1 in HPNE EVs and PANC-1 EVs, with flotillin-1 used as an EV-associated reference marker. (b) Representative AFM-based recognition maps acquired over 100 nm × 100 nm EV surface regions for ITG, EpCAM, and GPC1 on PANC-1 EVs and HPNE EVs. Colored pixels indicate accepted ligand-specific rupture events detected during force mapping. Color scales indicate rupture force in pN. (c) Apparent recognition probability calculated from individual EV maps for each biomarker/EV condition. Each dot represents one EV map used for recognition-probability analysis, and bars indicate mean ± SD. (d–f) Violin/scatter plots of accepted single-rupture forces measured at a pulling speed of 1 µm s^−1^ for (d) ITG–cRGD, (e) EpCAM–antibody, and (f) GPC1–antibody interactions on PANC-1 EVs and HPNE EVs. Each dot represents one accepted single-rupture event. Statistical comparisons were performed between PANC-1 EVs and HPNE EVs for each biomarker using a two-sided Mann–Whitney test (*p < 0.05; ***p < 0.001).

AFM-based recognition maps were then used to evaluate the functional accessibility of these EV surface targets at the single-vesicle level (Fig. 3b and c). The representative maps in Fig. 3b were acquired over 100 nm × 100 nm EV surface regions, where colored pixels indicate local probe–EV contacts that produced accepted ligand-specific rupture events. Because EV membrane proteins may undergo lateral diffusion during measurement^23^ and the probe samples a finite contact area, the maps provide functional recognition readouts, and the resulting event fractions were reported as apparent recognition probabilities.

The apparent recognition probabilities of ITG, EpCAM, and GPC1 were 28.6 ± 3.6%, 10.6 ± 2.9%, and 18.8 ± 3.0% for PANC-1 EVs and 8.6 ± 2.4%, 10.0 ± 2.5%, and 10.4 ± 1.9% for HPNE EVs, respectively (Fig. 3c). Thus, PANC-1 EVs showed a markedly higher apparent recognition probability for ITG and a moderate increase for GPC1 compared with HPNE EVs, whereas EpCAM recognition was similar between the two EV preparations. All three markers remained detectable on HPNE EVs. These apparent recognition probabilities are qualitatively consistent with the ensemble western blot trends observed for the same EV preparations, while providing complementary single-vesicle information. Western blotting reports population-averaged protein abundance, whereas AFM force mapping reports ligand-accessible recognition events on individual EVs. Together with the specificity controls summarized in Fig. 2e and Fig. S3, these recognition maps support ligand-specific detection of ITG, EpCAM, and GPC1 on individual EVs.

The fixed-speed rupture-force distributions were then analyzed using accepted single-rupture events measured at a pulling speed of 1 µm s^−1^ (Fig. 3d–f). The violin/scatter plots show the full event-to-event variability of rupture forces for each biomarker/EV condition, with each dot representing one accepted single-rupture event. The distributions were asymmetric and skewed, consistent with the stochastic nature of single-molecule rupture under force. Therefore, the median rupture force is used to summarize the stochastic single-molecule rupture-force distribution. Under the common measurement conditions used here, ITG–cRGD, EpCAM–antibody, and GPC1–antibody interactions showed distinct rupture-force distributions and statistically significant differences between PANC-1 and HPNE EVs for each biomarker.

At the fixed pulling speed of 1 µm s^−1^, the median apparent rupture forces for ITG–cRGD, EpCAM–antibody, and GPC1–antibody interactions on PANC-1 EVs were 113, 82, and 116 pN, with corresponding values of 130, 96, and 130 pN on HPNE EVs, respectively (Fig. 3d–f). At this speed, ITG–cRGD and GPC1–antibody interactions showed higher median rupture forces than EpCAM–antibody interactions, and the median was higher for HPNE EVs than for PANC-1 EVs for each ligand–receptor pair. Because the cRGD peptide and antibody probes differ in linker mechanics, binding geometry, and effective loading rate, these fixed-speed measurements reflect their respective probe configurations. The DFS analysis in the next section considers loading-rate dependence when evaluating apparent force-dependent dissociation behavior.

### Dynamic force spectroscopy of EV biomarker–ligand interactions

Because rupture forces depend on both retraction velocity and effective stiffness, fixed-speed rupture-force distributions alone do not fully describe force-dependent bond stability. We therefore performed dynamic force spectroscopy (DFS) by varying the probe retraction speed *v* and calculating the nominal loading rate as *r* = *v*·〈*k*_eff_〉, where 〈*k*_eff_〉 is the mean of the local effective stiffness values obtained from the slopes immediately preceding rupture. These values showed no systematic dependence on *v* over the investigated range. Here, *k*_eff_ represents the effective stiffness of the complete cantilever–PEG–ligand/receptor–EV–PLL system (Fig. 4a). The distributions and corresponding loading rates are provided in Fig. S4 and Table S3, respectively. For each biomarker/EV/loading-rate condition, rupture-force distributions were fit with the Bell–Evans probability-density function to determine the most probable rupture force, *F*_*r*_, as illustrated by representative low- and high-loading-rate distributions for ITG–cRGD interactions on PANC-1 EVs (Fig. 4b and Fig. S5).

**Fig. 4:**
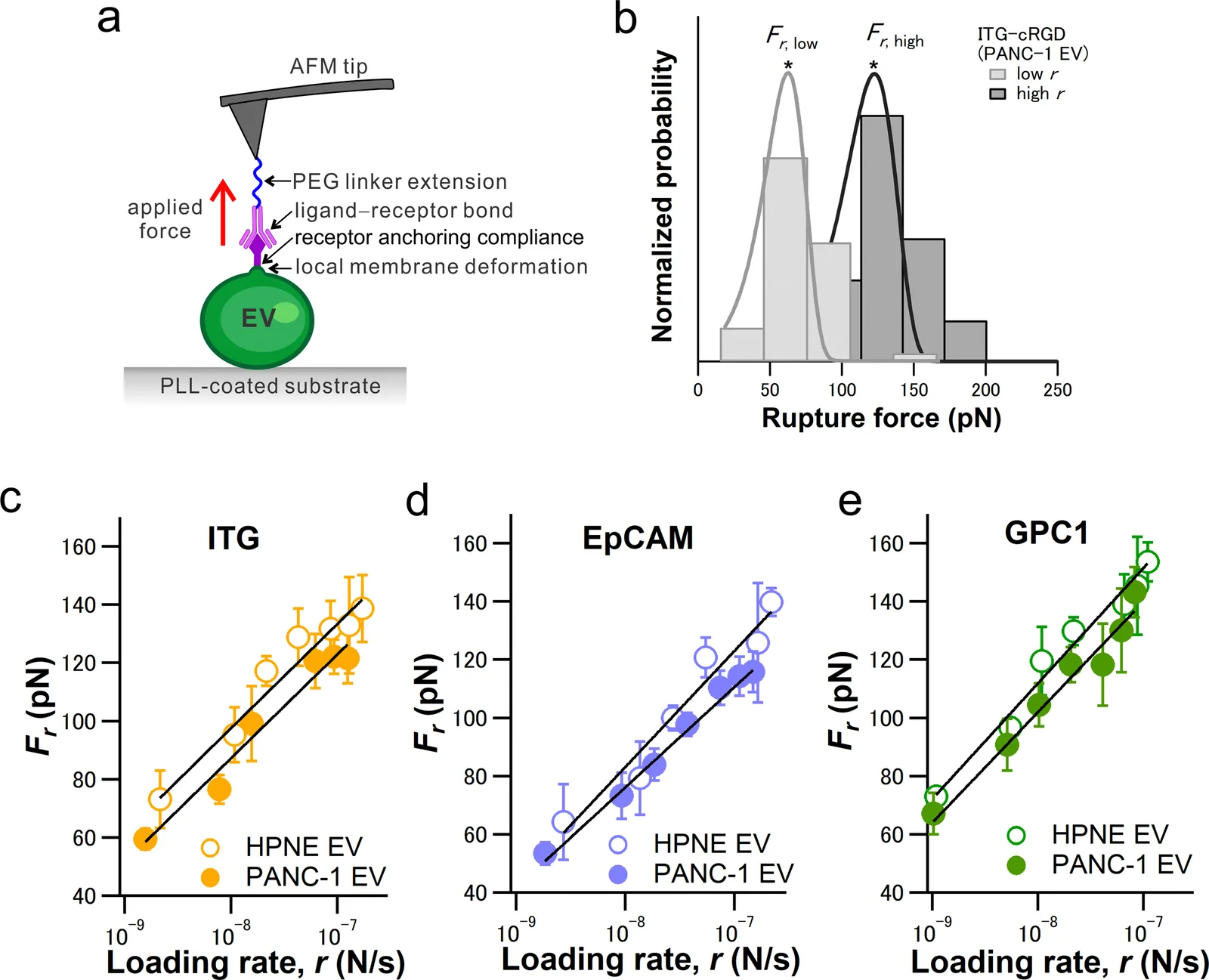
Dynamic force spectroscopy and apparent Bell–Evans analysis of EV biomarker–ligand interactions. (a) Schematic illustrating contributions from PEG linker extension, receptor anchoring compliance, local EV membrane deformation, the PLL-supported interface, and ligand–receptor bond rupture to the measured response. (b) Representative normalized rupture-force distributions for ITG–cRGD interactions on PANC-1 EVs measured at low (1564 pN s^−1^) and high (93 840 pN s^−1^) loading rates. Solid curves show Bell–Evans probability–density fits, and asterisks indicate the fitted most-probable rupture forces, *F*_*r*_. (c–e) Dependence of the most probable rupture force *F*_*r*_ on loading rate *r* for (c) ITG–cRGD, (d) EpCAM–antibody, and (e) GPC1–antibody interactions on PANC-1 EVs and HPNE EVs. The *x*-axis is shown on a logarithmic scale, and solid lines represent Bell–Evans fits.

For all three ligand–receptor pairs and both EV origins, *F*_*r*_ increased with loading rate, consistent with force-assisted dissociation under dynamic loading (Fig. 4c–e). The loading-rate dependence was analyzed using the Bell–Evans relationship:^24^1
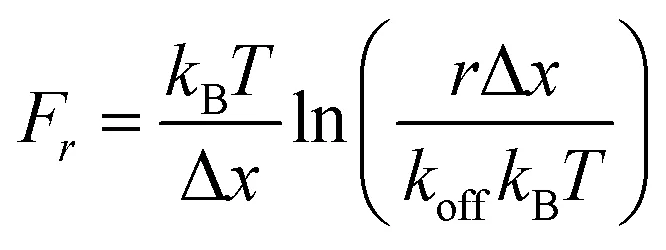
where Δ*x* is the effective distance to the transition state and *k*_off_ is the apparent force-free off-rate. Fitting *F*_*r*_ as a function of loading rate yielded apparent Bell–Evans parameters for each biomarker/EV combination. The corresponding apparent activation free-energy barrier, Δ*G*, was estimated from the Kramers relation,^25^

2 *k*_off_ = *ωe*^−Δ*G*/*k*_B_*T*^

using an attempt frequency of *ω* ≈ 5 × 10^9^ s^−1^ at 293 K (Table 1). At a fixed attempt frequency, Δ*G* is reported as a derived apparent quantity for comparison with previous work.

**Table 1:** Apparent single-molecule kinetic and thermodynamic parameters for receptor interactions

Biomarker–ligand	EV origin	*k*_off_ (s^−1^)	Δ*x* (nm)	Δ*G* (*k*_B_*T*)
EpCAM–Ab	PANC-1 EV	4.2 ± 2.5	0.27 ± 0.03	20.9 ± 0.6
EpCAM–Ab	HPNE EV	4.8 ± 2.9	0.23 ± 0.03	20.8 ± 0.6
ITG–cRGD	PANC-1 EV	2.4 ± 1.2	0.26 ± 0.02	21.5 ± 0.5
ITG–cRGD	HPNE EV	1.2 ± 1.5	0.26 ± 0.05	22.1 ± 1.2
GPC1–Ab	PANC-1 EV	1.0 ± 0.5	0.25 ± 0.02	22.3 ± 0.5
GPC1–Ab	HPNE EV	1.1 ± 1.1	0.23 ± 0.04	22.2 ± 1.0

Each Bell–Evans fit included six or seven loading-rate points and yielded *R*^*2*^ values of 0.951–0.991. The 95% joint confidence regions for apparent Δ*x* and ln(*k*_off_), calculated using the full slope–intercept covariance, are shown in Fig. S6. A covariance-weighted global fit with condition-specific intercepts and a shared slope yielded a common apparent Δ*x* of 0.254 ± 0.011 nm (95% CI: 0.231–0.276 nm). The six datasets were consistent with this shared-Δ*x* description (*Q* = 1.17, *df* = 5, *p* = 0.948). The shared-Δ*x* model indicates comparable apparent force sensitivity across the six conditions, while differences in apparent *k*_off_ primarily reflect condition-dependent offsets in the force–loading-rate relationships.

Across both EV origins, EpCAM–antibody interactions tended to show higher apparent *k*_off_ than ITG–cRGD and GPC1–antibody interactions. For PANC-1 EVs, the fitted point estimates suggested the order EpCAM, ITG, and GPC1, whereas for HPNE EVs, EpCAM was higher and the ITG and GPC1 estimates were nearly identical. These within-origin tendencies are reflected in the relative offsets of the force–loading-rate relationships in Fig. S5. Because several joint confidence regions overlapped (Fig. S6), these patterns are interpreted as trends rather than a fully resolved hierarchy. The observed condition dependence may reflect differences in ligand–receptor chemistry, linker mechanics, binding geometry, receptor organization, post-translational modification, receptor anchoring, and the local EV membrane environment.^26–28^

Parameters were obtained from Bell–Evans fits, with uncertainties estimated by first-order propagation including slope-intercept covariance.

The Bell–Evans parameters in Table 1 are reported as apparent, model-dependent quantities obtained under the present AFM–DFS conditions. This distinction is particularly important for *k*_off_, which depends exponentially on the fitted intercept-to-slope ratio and is sensitive to loading-rate range, slope–intercept covariance, event-selection criteria, linker mechanics, and EV membrane compliance. The sub-0.3 nm Δ*x* values are interpreted as apparent effective transition distances for the mechanically coupled probe–PEG–ligand/receptor–EV–PLL interface over the investigated loading-rate range. The extracted parameters provide a consistent basis for comparing ligand–receptor pairs measured using the same protocol.

As illustrated in Fig. 4a, the measured rupture response includes PEG linker extension, receptor anchoring compliance, local EV membrane deformation, the PLL-supported interface, and ligand–receptor bond rupture. Surface immobilization may influence EV membrane tension, receptor organization, and accessibility.^22^ All six conditions were evaluated using the same PLL preparation and measurement configuration. These coupled interfacial effects are relevant to EV capture and sensing under flow, washing, and repeated-contact conditions.

Comparison with our previous ITG–cRGD measurements on intact pancreatic cancer cell membranes further illustrates the importance of mechanical context. The EV measurements showed an apparent activation barrier comparable to the intact-cell value, together with a larger apparent Δ*x* and higher apparent *k*_off_.^15^ These differences may reflect contributions from the cytoskeleton-free EV membrane, receptor anchoring, and receptor mobility.^29,30^

Together, the DFS and recognition measurements show that ligand accessibility and apparent force-dependent stability are complementary properties of EV affinity interfaces. Ligand selection for capture and sensing should therefore consider both recognition probability and mechanical stability under the operating conditions of the interface. Interactions with rapid apparent dissociation may benefit from multivalent presentation, optimized surface density, or gentler washing conditions.

## Conclusions

In this study, we combined EV characterization, AFM-based single-molecule force spectroscopy, and dynamic force spectroscopy to examine ligand–receptor mechanics at extracellular vesicle biointerfaces. Using EVs derived from PANC-1 and HPNE cell models, we compared three PDAC-associated EV surface targets, ITGαvβ5, EpCAM, and GPC1. PANC-1 EVs showed higher particle concentration and stronger ITG and GPC1 expression than HPNE EVs, while AFM force mapping revealed higher apparent ligand-recognition probability for ITG and GPC1 and similar EpCAM recognition between the two EV preparations. These results distinguish population-level biomarker expression from functional ligand accessibility on individual EVs.

Analysis of accepted single-rupture events and the DFS data further revealed interaction-dependent differences in apparent rupture behavior. The six conditions showed comparable apparent force sensitivity, while condition-dependent offsets in their force–loading-rate relationships corresponded to differences in apparent dissociation behavior. EpCAM–antibody interactions tended to show higher apparent *k*_off_ than ITG–cRGD and GPC1–antibody interactions, while ITG and GPC1 were less clearly separated. Comparison with previous ITG–cRGD measurements on intact pancreatic cancer cell membranes also suggests that the compliant, cytoskeleton-free EV membrane environment contributes to the apparent rupture response. Together with the recognition measurements, these results demonstrate that biomarker accessibility, rupture mechanics, and membrane context provide complementary information about EV surface targeting under force.

Future studies incorporating additional EV models, patient-derived samples, and orthogonal ensemble kinetic methods such as SPR or BLI will help relate apparent single-molecule rupture parameters to multivalent EV binding under practical capture conditions. These findings support the use of single-molecule force spectroscopy alongside receptor-expression profiling in the design of EV capture, sensing, and affinity-interface platforms.

## Experimental

### Cell culture and extracellular vesicle (EV) isolation

PANC-1 and hTERT–HPNE cell lines were obtained from the American Type Culture Collection and maintained as described previously.^5,15^ Briefly, cells were cultured at 37 °C in a humidified incubator with 5% CO_2_ and 95% air. PANC-1 cells were grown in high-glucose Dulbecco's modified Eagle's medium (DMEM) supplemented with 10% fetal bovine serum (FBS) and 1% penicillin–streptomycin. hTERT–HPNE cells were cultured in high-glucose DMEM supplemented with 10% FBS, 1% penicillin–streptomycin, and 0.75 µg mL^−1^ puromycin. EVs were isolated from conditioned media by differential ultracentrifugation.^5^ When cells reached sub-confluence, they were washed with PBS and the medium was replaced with medium containing 10% exosome-depleted FBS (Gibco, A2720801). After 48 h, conditioned media were collected and centrifuged at 800×*g* for 5 min and 2000×*g* for 10 min to remove cells and debris, followed by filtration through 0.22 µm filters. The clarified supernatant was ultracentrifuged at 28 000 rpm overnight using an SW 28 Ti swinging-bucket rotor (Beckman Coulter) to pellet EVs. The supernatant was carefully aspirated, and EV pellets obtained from 30 mL of conditioned medium were resuspended in 200–500 µL PBS for subsequent analyses. Because differential ultracentrifugation does not resolve small EV subtypes such as exosomes and microvesicles, we refer to all pelleted vesicles operationally as EVs in line with current MISEV recommendations. Our goal here is to quantify receptor–ligand mechanics on the vesicle population obtained under these standardized isolation conditions rather than to distinguish specific EV subpopulations.

### Nanoparticle tracking analysis

The size distribution and concentration of EVs were measured by nanoparticle tracking analysis (NTA) using a NanoSight NS300 system (Malvern Panalytical, UK). EV samples were diluted ∼1000-fold in PBS and introduced into the flow cell using a syringe pump at a constant flow rate of 20 (instrument units). The instrument was equipped with a 532 nm laser and a high-sensitivity scientific CMOS camera. For each sample, at least three 30 s videos were recorded at 25 °C with a camera level of 11–13. Data were analyzed using NTA software (version 3.4) with a detection threshold of 4–6.

### EV preparation for imaging and AFM force measurements

For substrate preparation, cleaned silicon (SiO_2_) or freshly cleaved mica was incubated for 90 s with 0.001% (w/v) poly-l-lysine (Sigma-Aldrich, P4707, molecular weight 70 000–1 50 000), followed by gentle rinsing with water and brief drying under a nitrogen stream to remove excess liquid. Apparent PLL thickness under ambient conditions was determined from height profiles across locally cleared coating boundaries on identically prepared SiO_2_ substrates. For EV immobilization, a 10–20 µL droplet of EV suspension in PBS was placed on the PLL-coated substrate and incubated for 60 min at 4 °C. The samples were then gently rinsed with PBS to remove unbound vesicles. For dry imaging, substrates were gently dried under a nitrogen stream and imaged by atomic force microscopy (AFM; NTEGRA, NT-MDT) or scanning electron microscopy (SEM; JSM-7600F, JEOL). AFM images were typically acquired at 256 × 256 pixels with a scan rate of 0.5–1 Hz, depending on scan size and sample morphology. Images were flattened, when necessary, to remove background tilt and low-frequency noise by applying least-squares polynomial fits to unmasked scan lines. For single-molecule force spectroscopy (SMFS) and dynamic force spectroscopy (DFS), EVs were maintained in PBS after rinsing, and measured without an intervening drying step.

### Western blotting

EVs were lysed in NP40 cell lysis buffer (Invitrogen) supplemented with PMSF and 1× Halt protease and phosphatase inhibitor cocktail (Thermo Fisher Scientific). Protein concentrations were determined using the Pierce BCA Protein Assay Kit (Thermo Fisher Scientific), and equal protein amounts were loaded in each lane. Proteins were separated by SDS–PAGE using Mini-PROTEAN TGX precast gels (Bio-Rad) and transferred to nitrocellulose membranes using a Trans-Blot Turbo transfer system (Bio-Rad). Membranes were blocked with 5% non-fat dry milk in TBST (TBS + 0.1% Tween-20) for 1 h at room temperature and then incubated overnight at 4 °C with primary antibodies: ITGαv (ab179475, Abcam, 1 : 5000), ITGβ5 (ab184312, Abcam, 1 : 1000), GPC1 (PA528055, Thermo Scientific, 1 : 2000), EpCAM (ab71916, Abcam, 1 : 1000), CD9 (ab223052, Abcam, 1 : 2000), CD63 (ab216130, Abcam, 1 : 1000), and Flotillin-1 (ab133497, Abcam, 1 : 1000). After washing with TBST, membranes were incubated with the appropriate HRP-conjugated secondary antibodies (1 : 3000) for 1 h at room temperature. Following additional washing, chemiluminescent signals were developed using SuperSignal West Femto Maximum Sensitivity Substrate (Thermo Fisher Scientific). Images were acquired using a FluorChem FC2 imaging system (Alpha Innotech), with exposure times ranging from 30 s to 5 min depending on signal intensity, and analyzed using ImageJ. Full, uncropped chemiluminescence images corresponding to Fig. 1e, 3a, and Fig. S3a are provided in Fig. S7, S8, and S9, respectively.

### Synthesis and characterization of the cRGD peptide

The cRGD peptide was synthesized as described previously^15,31^ using microwave-assisted solid-phase peptide synthesis on a Liberty Blue synthesizer (CEM Corporation) with Rink amide resin. The sequence hexynoic acid–Cys(Acm)–Arg(Pbf)–Gly–Asp(OBtu)–Lys(Boc)–Gly–Pro–Asp(OBtu)–Cys(Acm)–NH2 was assembled under standard Fmoc chemistry without final side-chain deprotection. Cyclization was achieved by treating the peptide–resin conjugate with thallium trifluoroacetate in DMF, followed by washing and cleavage from the resin with a TFA/water/triisopropylsilane cocktail. The crude peptide was precipitated with cold diethyl ether, collected, dried under vacuum, and used without further purification. The product was characterized by MALDI-ToF mass spectrometry as shown in Fig. S1.

### AFM probe functionalization with cRGD peptide or antibodies

AFM probes were functionalized with either cRGD peptide or antibodies using previously reported coupling strategies.^15,19,20^ Briefly, silicon nitride tips were washed with chloroform, dried under nitrogen, and treated in a UV–ozone cleaner. Tips were then immersed overnight at room temperature in an ethanolamine hydrochloride/DMSO solution (3.3 g ethanolamine hydrochloride in 6 mL DMSO) to introduce amino groups, followed by rinsing with DMSO and ethanol and drying under nitrogen. For cRGD functionalization, amino-functionalized tips were incubated for 2 h in chloroform containing Azido–PEG_12_–NHS ester (BP-21607, BroadPharm; MW 740.8; 12 ethylene oxide units; nominal fully extended PEG length approximately 5 nm; 6.6 mg mL^−1^), rinsed extensively with chloroform, and dried. Tips were then immersed in 100 µL DMSO containing 1 mg cRGD peptide, followed by addition of 900 µL deionized water. Copper complex catalyst and ascorbate solutions (16 µL each) were added, and the Cu(i)-catalyzed azide–alkyne cycloaddition (“click” reaction) was allowed to proceed for 24 h at room temperature with gentle rocking. Functionalized tips were rinsed with PBS before use. For antibody functionalization (EpCAM or GPC1), amino-functionalized tips were incubated for 2 h in 0.5 mL chloroform containing Acetal–PEG–NHS (PJK-1701, Creative PEGWorks; nominal PEG MW 1 kDa; approximately 23 ethylene oxide units; nominal fully extended PEG length approximately 8 nm; 1 mg mL^−1^) and 0.5% (v/v) TEA to attach acetal–PEG linkers, followed by extensive rinsing with chloroform. Acetal groups were converted to aldehydes by immersion in acetone containing 0.1% iodine for 5 min and subsequent rinsing in pure acetone. Tips were then incubated for 1 h in 50 µL antibody solution (0.2 mg mL^−1^ in PBS) supplemented with 2 µL 1 M NaCNBH_3_ to stabilize Schiff-base linkages *via* reductive amination. Finally, 5 µL ethanolamine hydrochloride (1 M, pH 9.6) was added and incubation continued for 10 min to cap remaining aldehydes. Tips were rinsed with PBS and stored in PBS at 4 °C until use (up to several days). Matched linker-only control probes were prepared for both tether architectures. Azido–PEG_12_ control probes underwent the complete click-reaction procedure without cRGD. Acetal–PEG control probes underwent aldehyde conversion and incubation with NaCNBH_3_ in the absence of antibody, followed by ethanolamine capping. Control measurements were performed on PANC-1 EVs using the same force-mapping and event-selection procedures as the ligand-functionalized probes.

### Force spectroscopy measurements

Force spectroscopy measurements were performed using an AFM (NTEGRA, NT-MDT) and V-shaped silicon nitride probes (PNP-TR, Nanoworld) with a nominal spring constant of 0.08 N m^−1^ and a face angle of 35°. The cantilever spring constant was calibrated using the thermal noise fluctuation method, and the deflection sensitivity of each probe was determined from force–distance curves acquired on an exposed rigid region on the substrate. For EV measurements, the indentation depth was limited to approximately 5–10% of the apparent height of the immobilized EV to minimize additional probe-induced deformation after immobilization. Measurements were performed as force maps over selected EV regions, and repeated indentation at the same lateral position was avoided when possible. For rupture-force analysis, accepted single-rupture events were defined as force–distance curves containing one discrete, well-defined rupture step after tip–EV contact. Consistent selection criteria were applied across pulling speeds based on rupture position relative to contact, event morphology, baseline stability, and background-control behavior. Curves showing visibly multiple rupture steps, broad adhesion, long or tether-like extensions, poorly defined rupture features, or complex patterns were excluded. Force magnitude was not used as a standalone exclusion criterion. Representative excluded curves and contact-to-rupture separation statistics are provided in Fig. S2 and Table S1, respectively. Background and nonspecific interactions were evaluated using bare-tip, matched linker-only, and competitive-blocking controls. Apparent recognition probability was calculated as the fraction of force curves containing accepted ligand-specific rupture events. Five EV maps were analyzed for each biomarker/EV condition. For the fixed-speed rupture-force distributions, 64–150 accepted events collected from multiple EVs were pooled for each condition. Distributions were summarized by the median and interquartile range, and PANC-1 and HPNE conditions were compared for each biomarker using a two-sided Mann–Whitney test. Possible multiple-bond contributions were estimated from the apparent recognition probabilities using the Poisson model described in the Results and Table S2. For DFS analysis, 28–162 accepted events per biomarker/EV/loading-rate condition were used to construct rupture-force distributions. Local effective stiffness values *k*_eff_ were obtained from the slopes of the retraction curves immediately preceding rupture. A condition-specific mean 〈*k*_eff_〉 calculated from measurements spanning the pulling-speed range was used to determine the nominal loading rate as *r* = *v* 〈*k*_eff_〉. Rupture-force distributions at each loading rate were fit with the Bell–Evans probability-density function to determine the most probable rupture force *F*_*r*_. Each *F*_*r*_*versus* ln *r* dataset was then fit by ordinary least-squares linear regression. Uncertainties in Δ*x* and *k*_off_ were propagated using the slope–intercept covariance matrices from the Bell–Evans fits. The 95% joint confidence regions were obtained by transforming the parameter covariance regions into (Δ*x*, ln(*k*_off_)) space. A generalized least-squares global model with condition-specific intercepts and a shared slope was used to evaluate a common apparent Δ*x*, and slope heterogeneity was assessed using the *Q* statistic.

## Author contributions

Study concept and design (S. T., Y. C.); data acquisition, analysis, or interpretation (S. T., R. J., S. H., C. E., Y. P. R. J., M. Q., S. M., J. K., Y. C.); the manuscript was written through the contributions of all authors. All authors have approved the final version of the manuscript.

## Conflicts of interest

There are no conflicts to declare.

## Supplementary Material

TB-014-D6TB01369D-s001

## Data Availability

The data supporting this paper are provided in the manuscript and supplementary information (SI). The SI includes characterization of the PLL-coated substrate; a MALDI-ToF mass spectrum obtained for the cRGD peptide preparation; fluorescence analysis of ligand coupling on Si_3_N_4_ substrates; representative excluded force–distance curves and contact-to-rupture separation statistics from accepted curves; relative integrin protein expression in knockdown EVs and recognition maps obtained using blocking, knockdown, bare-tip, and matched linker-only controls; Poisson-model estimates of single- and multiple-bond fractions; representative local effective-stiffness distributions, condition-specific mean *k*_eff_ values used in the DFS analysis, and corresponding loading-rate ranges; additional rupture-force probability-density fits; and covariance-based 95% joint confidence regions for the Bell–Evans parameters. Full, uncropped chemiluminescence images underlying the Western blots presented in Fig. 1e, 3a, and Fig. S3a are provided in Fig. S7, S8, and S9, respectively. See DOI: https://doi.org/10.1039/d6tb01369d.
