# Supplementary material for: Single-molecule force spectroscopy of ligand–receptor mechanics at extracellular vesicle biointerfaces
Source: J Mater Chem B. 2026 Sep 30;14(42):13092–101. doi: 10.1039/d6tb01369d (PMC13643719; doi:10.1039/d6tb01369d)
Supplement: Supplementary file 2 [file TB-014-D6TB01369D-s001.pdf]

Supplementary Information (SI) for Journal of Materials Chemistry B.
This journal is © The Royal Society of Chemistry

Supplementary Information

# Single-Molecule Force Spectroscopy of Ligand-Receptor Mechanics at Extracellular Vesicle Biointerfaces

*Sakurako Tani,[a] Reed Jacobson,[b] Sangdeuk Ha,[b] Connor Edvall,[c] Yagna P. R. Jarajapu,[c] Mohiuddin Quadir,[de] Sanku Mallik,[c] Jiha Kim,[bf] and Yongki Choi*[*aef]*

Departments of Physics,[a] Biological Sciences,[b] Pharmaceutical Sciences,[c] Coatings and Polymeric Materials,[d] Materials and Nanotechnology Program,[e] and Molecular and Cellular Biology Program,[f] North Dakota State University, Fargo, ND 58108, USA

[*]Email: yongki.choi@ndsu.edu

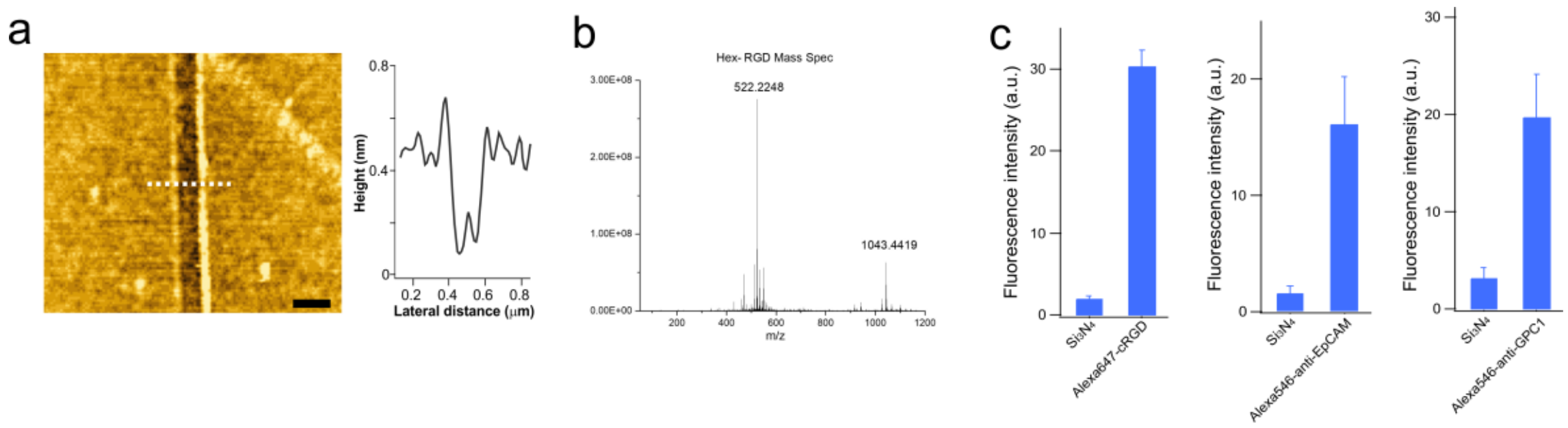


Figure S1. Characterization of the PLL coating, cRGD peptide, and ligand-functionalized surfaces. (a) Representative AFM topography of an identically prepared PLL-coated $SiO_2$ substrate containing a locally cleared region and corresponding height profile across the coating boundary. The dotted line indicates the profile location. Scale bar = 300 nm. (b) MALDI-ToF mass spectrum obtained for the cRGD peptide preparation. (c) Relative fluorescence intensities measured on bare $Si_3N_4$ substrates and substrates prepared using the complete PEG–azide or PEG–aldehyde coupling chemistry and incubated with Alexa647–RGD, Alexa546–anti-EpCAM, or Alexa546–anti-GPC1. Fluorescence measurements confirmed surface coupling. Tether density at the AFM tip apex was not quantified.

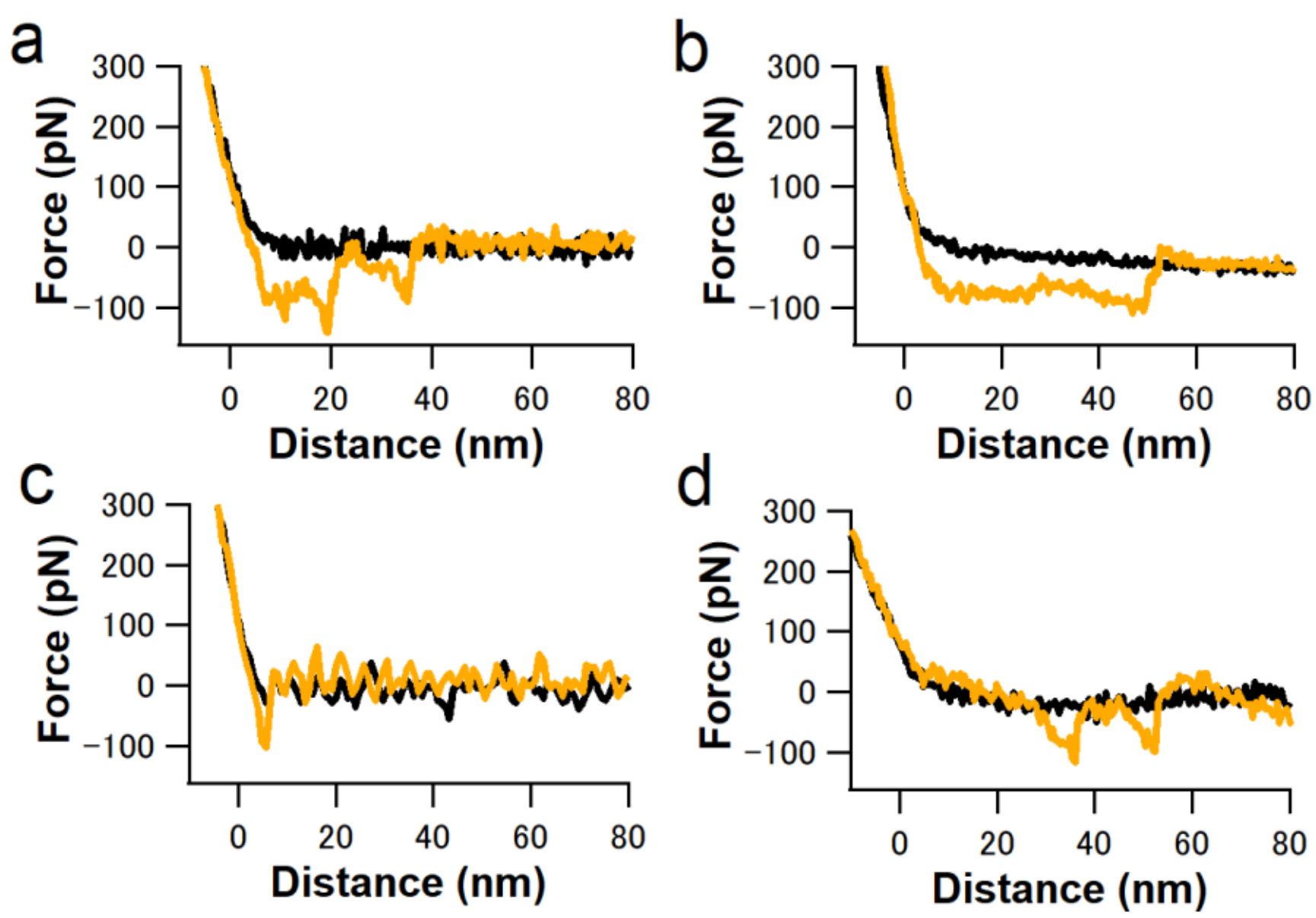


Figure S2. Representative force–distance curves excluded from single-rupture analysis. Black and orange traces represent approach and retraction curves, respectively. Examples include curves with (a) multiple rupture steps, (b) broad adhesive features or long/tether-like extensions, (c) unstable baseline or poorly defined rupture features, and (d) complex rupture patterns. Low-force events were not excluded based on force magnitude alone; event shape, separation distance, baseline stability, and consistency with background-control behavior were also considered.

Table S1. Apparent rupture separations.

| EV source | Interaction | Median [IQR], nm | Observed range, nm |
|---|---|---|---|
| PANC-1 | ITG-cRGD | 29.7 [26.0 - 31.0] | 25.1 - 33.5 |
| HPNE | ITG-cRGD | 24.4 [23.6 - 25.6] | 22.6 - 27.3 |
| PANC-1 | EpCAM-antibody | 35.9 [28.2 - 42.9] | 25.6 - 52.1 |
| HPNE | EpCAM-antibody | 32.0 [27.4 - 40.1] | 17.5 - 50.0 |
| PANC-1 | GPC1-antibody | 34.6 [33.3 - 47.4] | 26.4 - 51.7 |
| HPNE | GPC1-antibody | 40.3 [37.9 - 41.9] | 36.3 - 55.3 |

Apparent rupture separations from representative accepted force–distance curves spanning the DFS pulling-speed range ($n$ = 10 per condition) represent the contact-to-rupture distance of the complete probe–PEG–ligand/receptor–EV system.

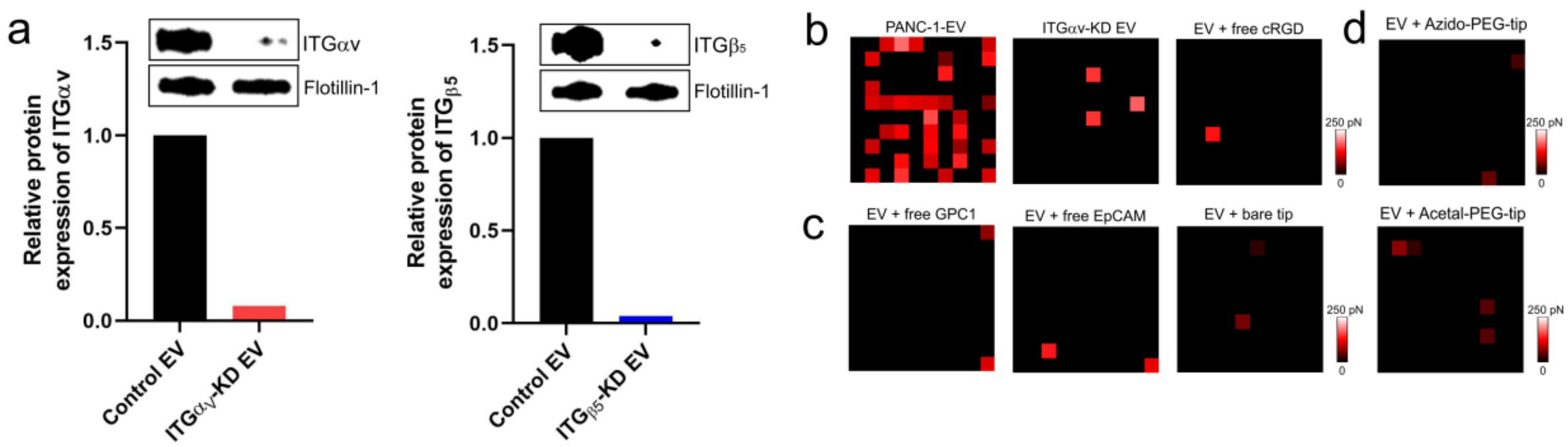


Figure S3. Representative specificity controls for AFM-based EV biomarker recognition. (a) Effective knockdown of integrin αv and β5 protein expression using lentiviral shRNA constructs,[1] as confirmed by immunoblotting of EVs isolated from integrin knockdown cells. (b) Recognition maps obtained with a cRGD-functionalized tip on control EVs, integrin αv-knockdown EVs, and EVs pre-incubated with free cRGD peptide. (c) Maps of EVs pre-incubated with free GPC1 or EpCAM antibody and probed with the corresponding antibody-functionalized tip, and a map of EVs probed with a non-functionalized tip. (d) Maps on EVs using Azido-$PEG_{12}$ linker-only and ethanolamine-capped Acetal-PEG linker-only probes. Each map area is 100 nm × 100 nm.

Table S2. Poisson estimates of single- and multiple-bond formation.

| EV source | Interaction | $p$, % | $\lambda$ | $P(N \geq 2)$, % | $P(N \geq 2 \mid N \geq 1)$, % | $P(N = 1 \mid N \geq 1)$, % |
|---|---|---|---|---|---|---|
| PANC-1 | ITG-cRGD | 28.6 | 0.337 | 4.55 | 15.9 | 84.1 |
| HPNE | ITG-cRGD | 8.6 | 0.090 | 0.38 | 4.4 | 95.6 |
| PANC-1 | EpCAM-antibody | 10.6 | 0.112 | 0.58 | 5.5 | 94.5 |
| HPNE | EpCAM-antibody | 10.0 | 0.105 | 0.52 | 5.2 | 94.8 |
| PANC-1 | GPC1-antibody | 18.8 | 0.208 | 1.89 | 10.1 | 89.9 |
| HPNE | GPC1-antibody | 10.4 | 0.110 | 0.56 | 5.4 | 94.6 |

The apparent recognition probability $p$ was used as an estimate of $P(N \geq 1)$. Assuming independent Poisson bond formation, $\lambda = -\ln(1 - p)$, $P(N \geq 2) = 1 - e^{-\lambda}(1 + \lambda)$, and $P(N \geq 2 \mid N \geq 1) = P(N \geq 2)/p$. Here, $P(N \geq 2)$ represents the estimated fraction of all tip–EV contacts involving two or more bonds, whereas $P(N \geq 2 \mid N \geq 1)$ and $P(N = 1 \mid N \geq 1)$ represent the estimated multiple- and single-bond fractions, respectively, among interaction-positive contacts.

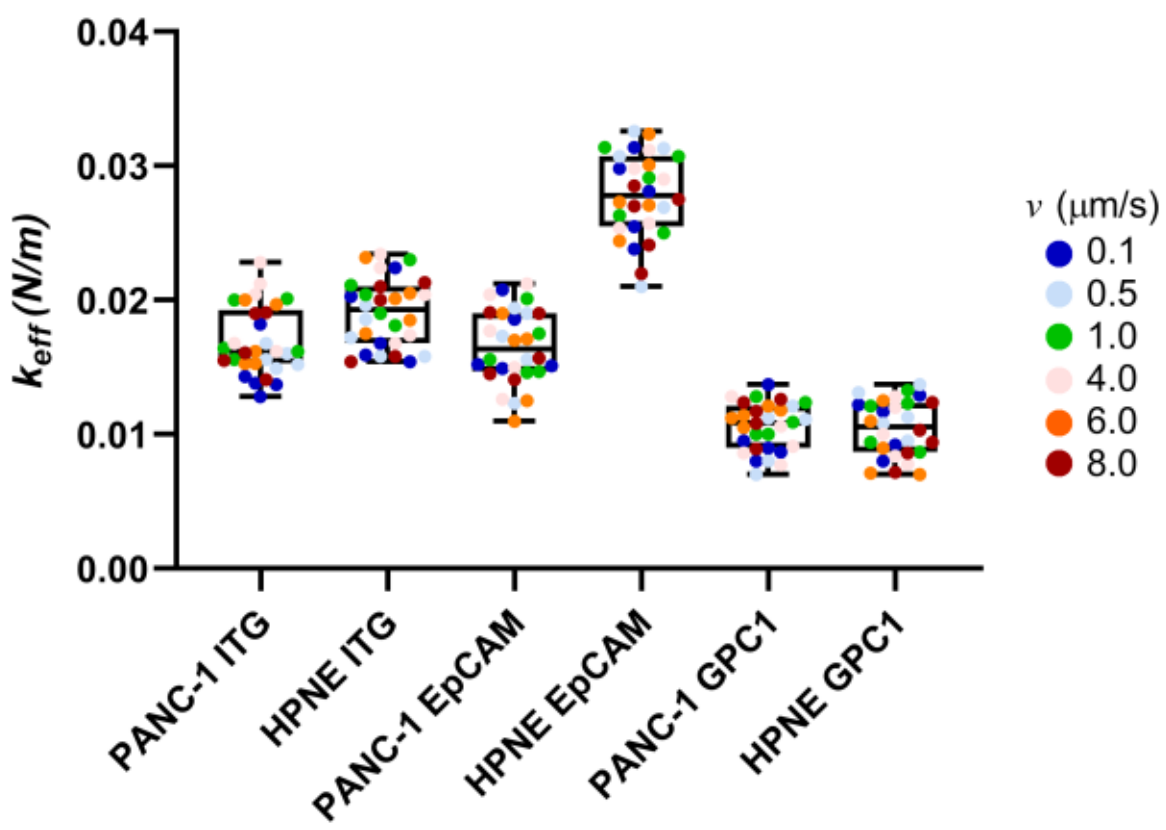


Figure S4. Representative distributions of local effective stiffness for the six biomarker/EV conditions (n = 30 measurements per condition; five measurements at each displayed pulling speed). Individual points are colored according to pulling speed; boxes show the median and interquartile range, and whiskers show the range. Values obtained at different pulling speeds overlapped without a consistent speed-dependent trend.

Table S3. Effective stiffness values and calculated loading-rate ranges.

| EV source | Interaction | $<k_{eff}>$ (N/m) | $r$ range (pN/s) |
|---|---|---|---|
| PANC-1 | ITG-cRGD | 0.0156 | $1.56 \times 10^3 – 1.25 \times 10^5$ |
| HPNE | ITG-cRGD | 0.0216 | $2.16 \times 10^3 – 1.73 \times 10^5$ |
| PANC-1 | EpCAM-antibody | 0.0185 | $1.85 \times 10^3 – 1.85 \times 10^5$ |
| HPNE | EpCAM-antibody | 0.0273 | $2.73 \times 10^3 – 2.19 \times 10^5$ |
| PANC-1 | GPC1-antibody | 0.0103 | $1.03 \times 10^3 – 8.21 \times 10^4$ |
| HPNE | GPC1-antibody | 0.0109 | $1.09 \times 10^3 – 1.09 \times 10^5$ |

Loading rates were calculated as $r = v \cdot <k_{eff}>$ over the pulling-speed range of 100–8,000 nm/s; PANC-1 EpCAM and HPNE GPC1 included an additional measurement at 10,000 nm/s.

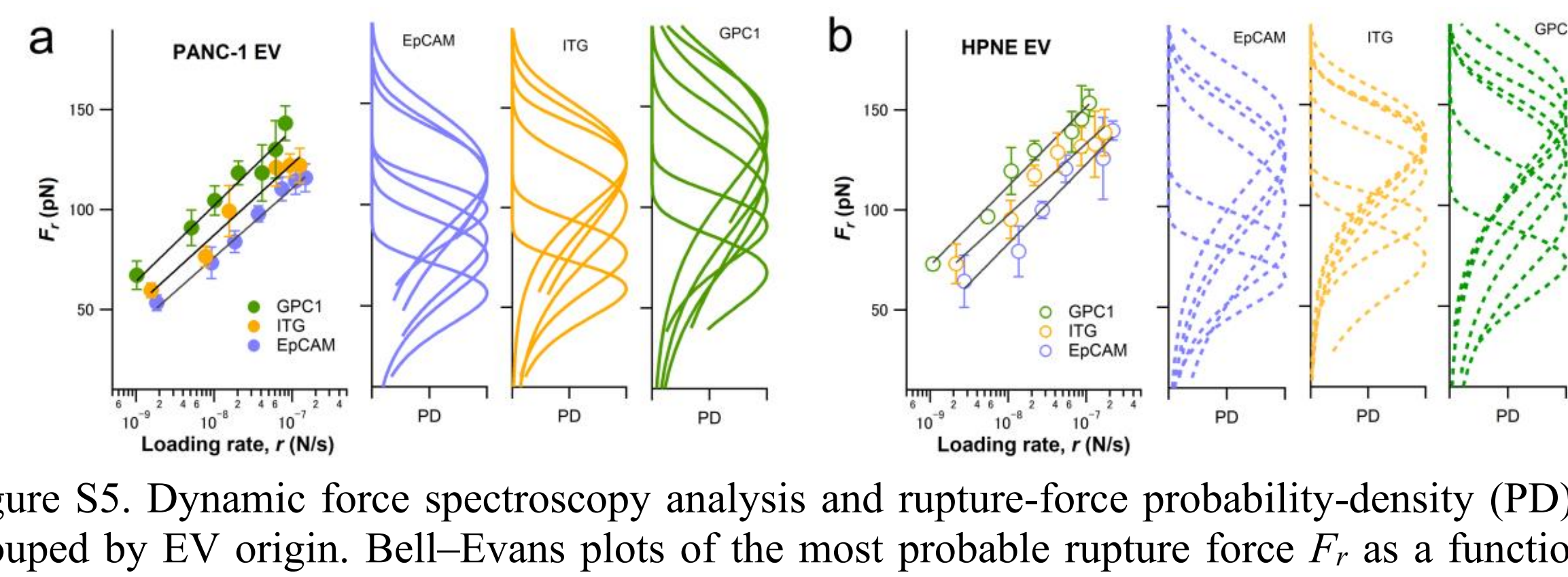


Figure S5. Dynamic force spectroscopy analysis and rupture-force probability-density (PD) fits grouped by EV origin. Bell–Evans plots of the most probable rupture force $F_r$ as a function of loading rate $r$ for ITG–cRGD, EpCAM–antibody, and GPC1–antibody interactions on (a) PANC-1 EVs and (b) HPNE EVs. Solid lines represent Bell–Evans fits. Corresponding rupture-force probability-density fits at different loading rates are shown to the right of each plot for EpCAM, ITG, and GPC1. These probability-density fits were used to determine the most probable rupture forces $F_r$ used in the DFS analysis.

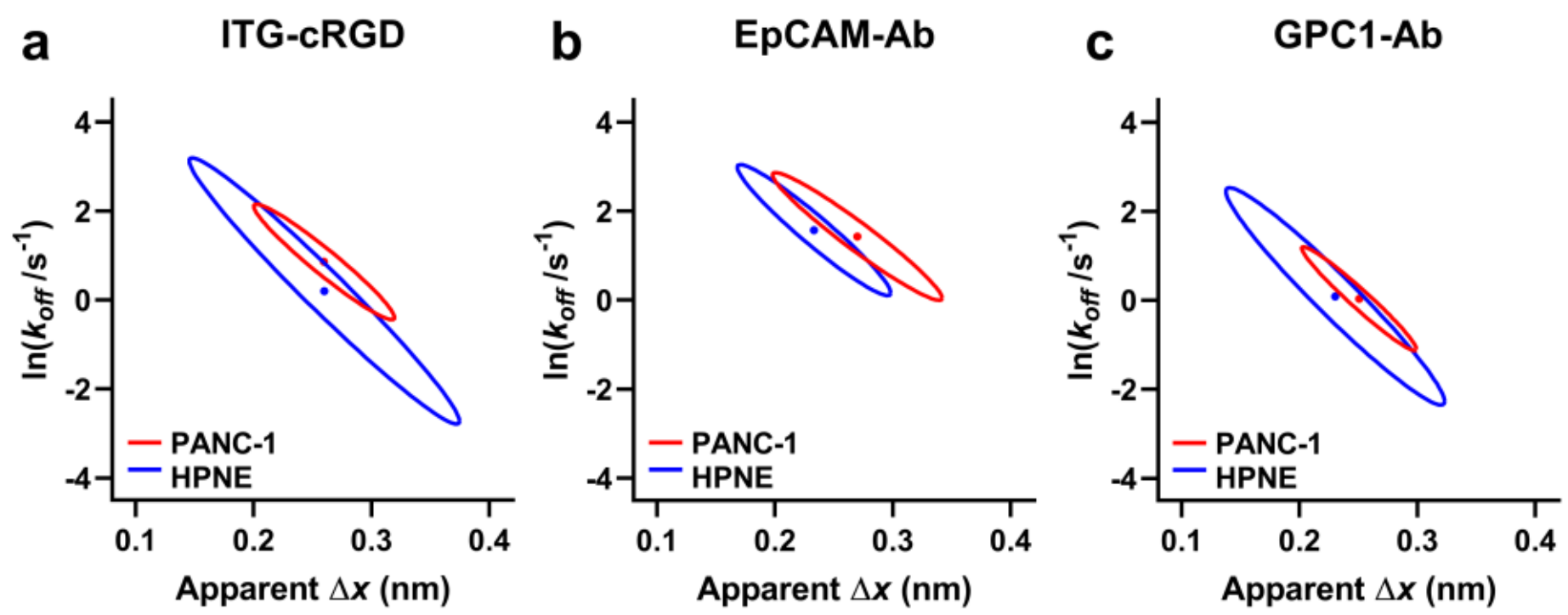


Figure S6. 95% joint confidence regions for apparent $\Delta x$ and $\ln(k_{off}/s^{-1})$ from the Bell–Evans fit. Red and blue regions represent PANC-1 and HPNE EVs, respectively, and the symbols indicate the best-fit parameter estimates. The confidence regions were calculated using the full covariance between the fitted slope and intercept.

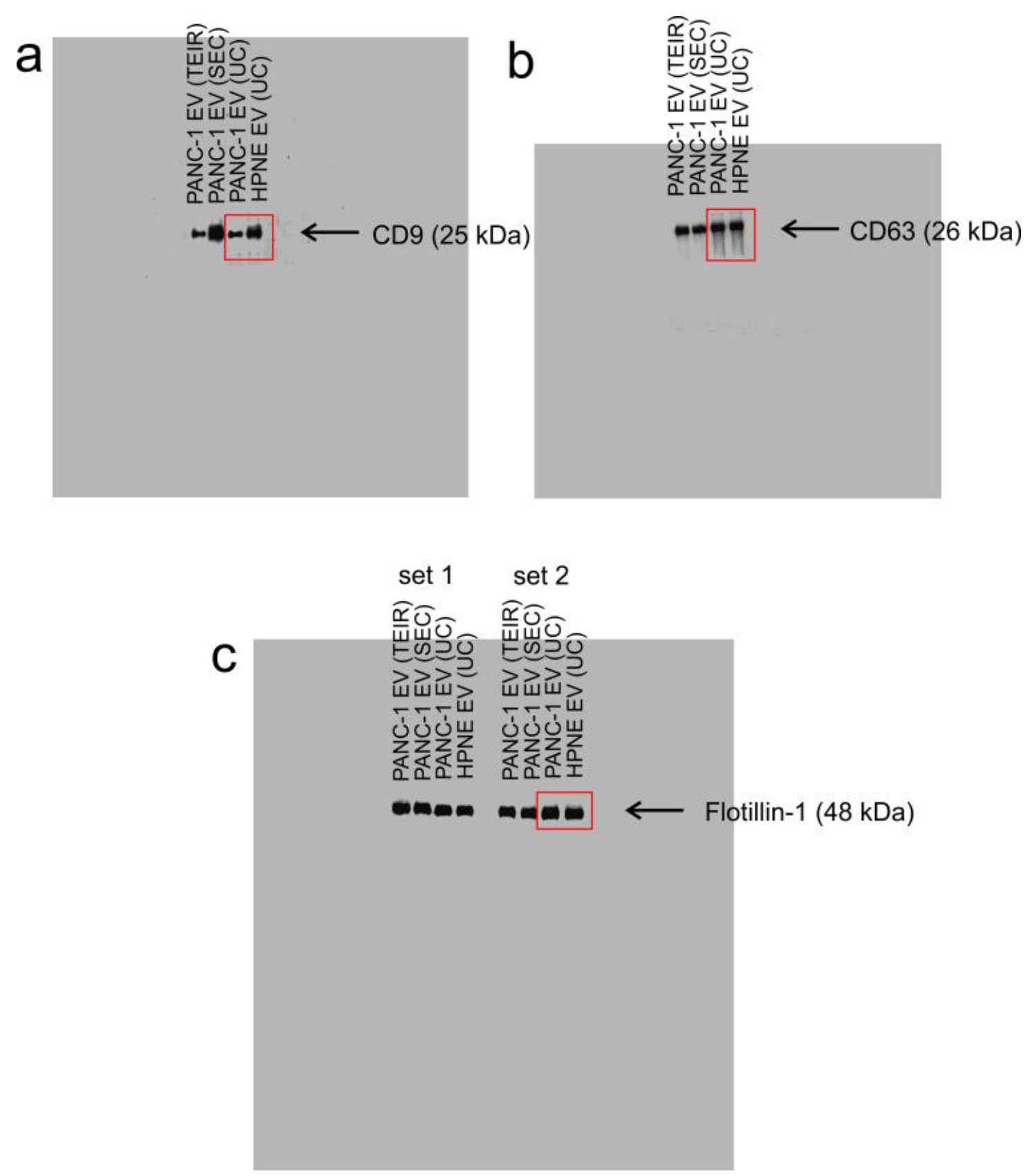


Figure S7. Uncropped Western blot images corresponding to Figure 1e. Full chemiluminescence images of the EV markers (a) CD9, (b) CD63, and (c) Flotillin-1 are shown. Red rectangles identify the lanes presented in Figure 1e. Lane identities are indicated above each blot. The Flotillin-1 image contains two repeated four-lane EV sample sets, both of which are shown for completeness. Molecular-weight markers are not detected in the archived chemiluminescence acquisitions; approximate expected molecular masses of the target proteins are indicated for reference. TEIR, total exosome isolation reagent; SEC, size-exclusion chromatography; UC, ultracentrifugation.

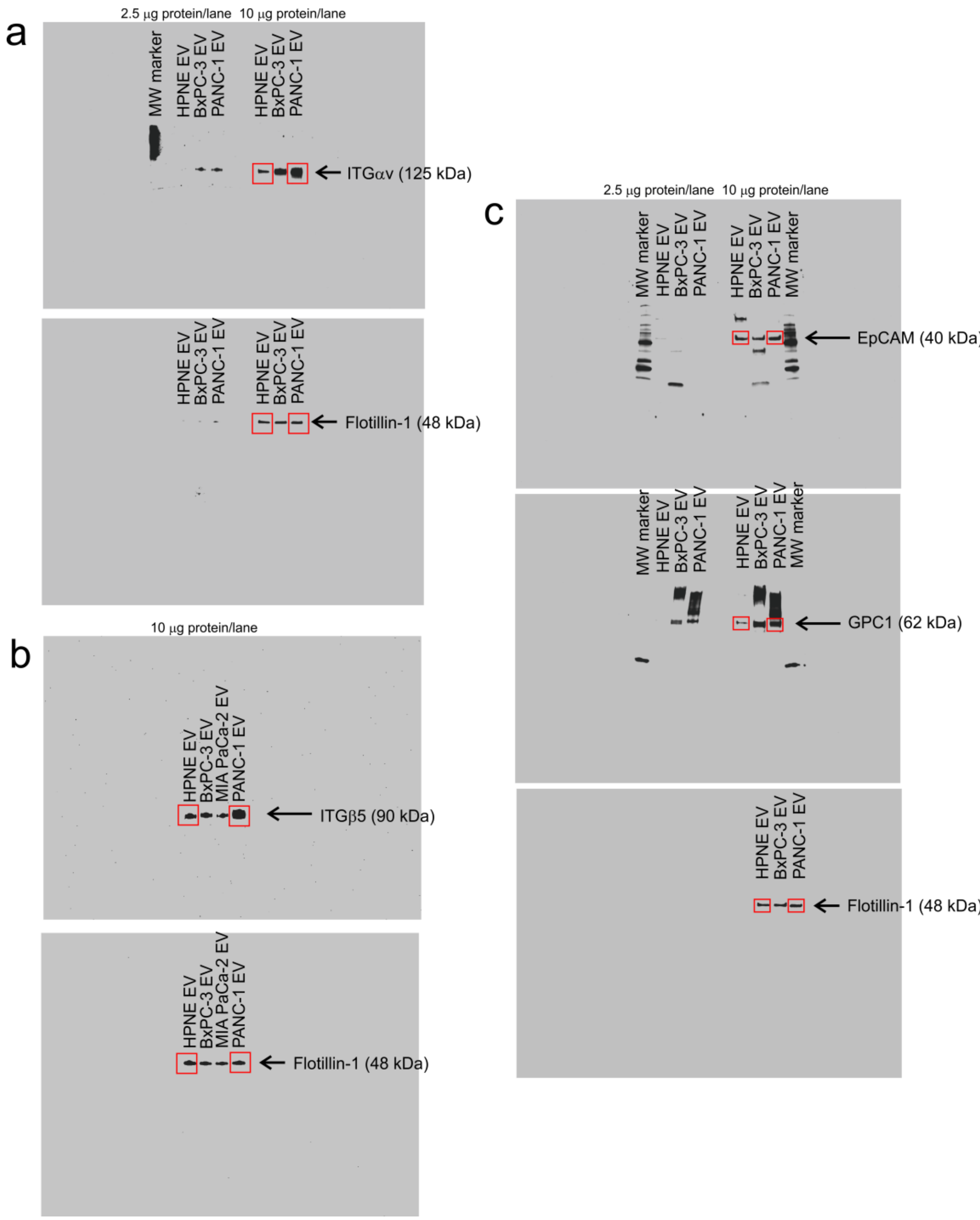


Figure S8. Uncropped Western blot images corresponding to Figure 3a. Full chemiluminescence images show (a) ITGαv and Flotillin-1, (b) ITGβ5 and Flotillin-1, and (c) EpCAM, GPC1, and Flotillin-1 in EV samples. Red rectangles identify the lanes presented in Figure 3a. Sample identities and protein amounts loaded per lane are indicated. Molecular-weight-marker lanes are labeled where visible, but individual marker masses were unavailable. Approximate expected molecular masses of the target proteins are indicated for reference. Non-adjacent HPNE and PANC-1 EV lanes from the respective blots were juxtaposed in Figure 3a.

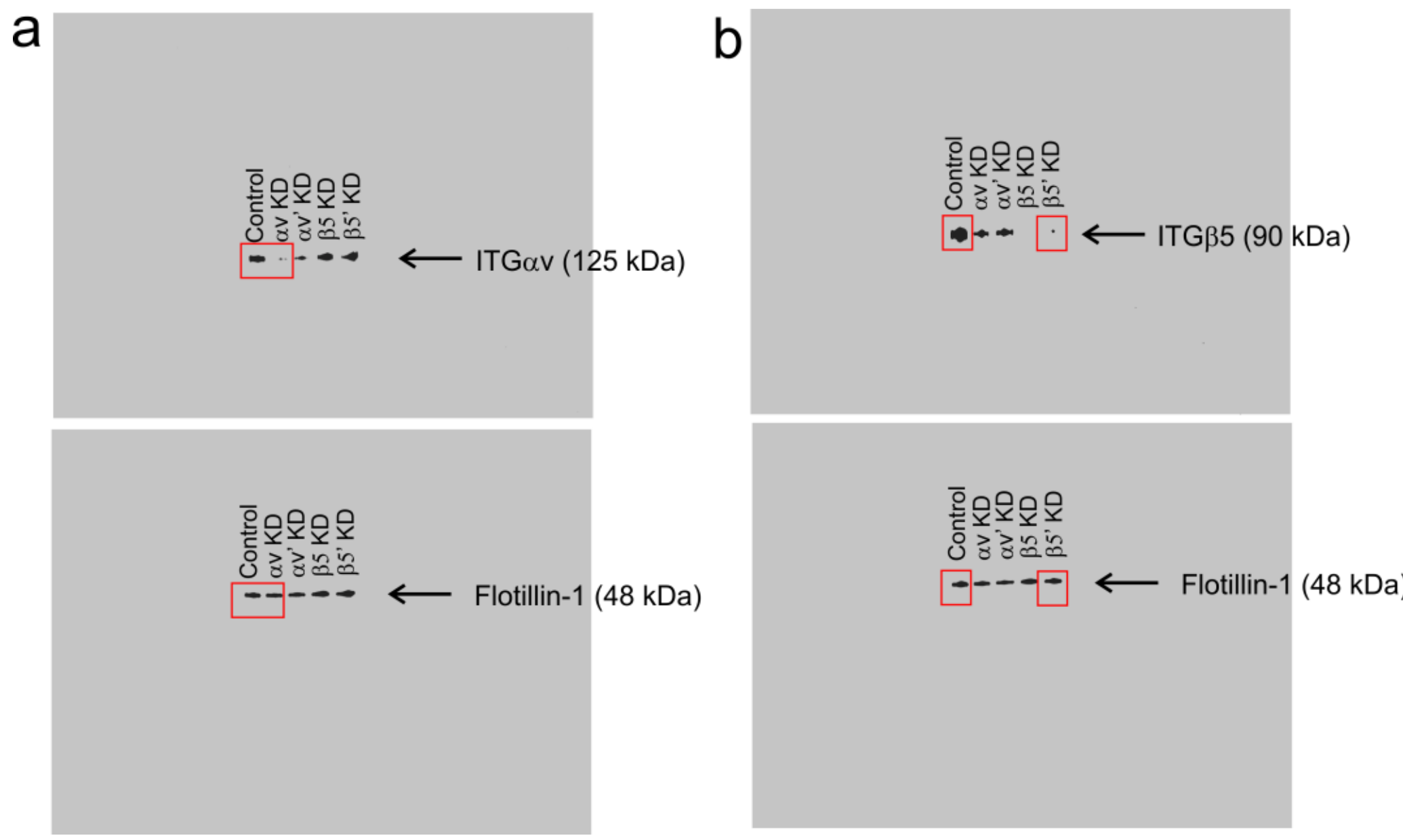


Figure S9. Uncropped Western blot images corresponding to Figure S3a. Full chemiluminescence images show (a) ITGαv and Flotillin-1 and (b) ITGβ5 and Flotillin-1 in EVs isolated from control and integrin-knockdown cells. Red rectangles identify the lanes presented in Figure S3a; non-adjacent lanes were juxtaposed in the cropped figure. Molecular-weight markers are not detected in the archived chemiluminescence acquisitions; approximate expected molecular masses of the target proteins are indicated for reference. αv KD and αv′ KD denote independent ITGαv-targeting shRNA constructs TRCN0000010768 and TRCN0000010769, respectively; β5 KD and β5′ KD denote independent ITGβ5-targeting shRNA constructs TRCN0000289121 and TRCN0000296117, respectively.

## References

(1) Alhalhooly, L.; Confeld, M. I.; Woo, S. O.; Mamnoon, B.; Jacobson, R.; Ghosh, S.; Kim, J.; Mallik, S.; Choi, Y. Single-Molecule Force Probing of RGD-Binding Integrins on Pancreatic Cancer Cells. *ACS Applied Materials & Interfaces* **2022**, *14* (6), 7671-7679. DOI: 10.1021/acsami.1c23361.
